# A Case of Solar Maculopathy Secondary to Reflected Sunlight During Garden Tiling

**DOI:** 10.7759/cureus.104010

**Published:** 2026-02-21

**Authors:** Nene Okamoto, Toshiyuki Oshitari, Tomohiko Usui

**Affiliations:** 1 Department of Ophthalmology, International University of Health and Welfare Narita Hospital, Narita, JPN; 2 Department of Ophthalmology and Visual Science, Chiba University Graduate School of Medicine, Chiba, JPN

**Keywords:** autofluorescence, ellipsoid zone, gardening, indirect sunlight, optical coherence tomography, solar maculopathy

## Abstract

Here, we present a rare case of solar maculopathy associated with repeated exposure to reflected sunlight without sunglasses during garden tiling. A 64-year-old woman presented with visual field defects during a comprehensive medical checkup and was referred to our hospital three years ago. She noticed mildly blurred vision and photophobia in both eyes. Fundus autofluorescence revealed ring-shaped hyperfluorescent lesions surrounding the fovea, and optical coherence tomography revealed disruption of the ellipsoid zones in the corresponding areas. Electroretinography showed no abnormalities, and the Goldmann visual field test showed mild enlargement of the Mariotte blind spot. She had engaged in gardening without wearing sunglasses for a month during the summer before her symptoms began. The patient was diagnosed with solar maculopathy on the basis of indirect sunlight exposure. As sunlight exposure is essential for diagnosing solar maculopathy, a detailed medical assessment is important for accurate diagnosis. This case may help raise awareness of the potential for solar maculopathy from indirect sunlight exposure during daily outdoor work.

## Introduction

Solar maculopathy usually occurs after exposure to sunlight or eclipses, resulting in photochemical damage to retinal pigment epithelial cells and photoreceptors [1-3]. Although some cases lack clear episodes of gazing directly at the sun [4,5], a clear history of sunlight exposure is usually required to diagnose solar maculopathy. Optical coherence tomography (OCT) reveals outer retinal changes in solar maculopathy, such as hyperreflectivity found in the en face outer retinal OCT images, and outer retinal holes and disruption of the ellipsoid zones found in the B-scan images [6,7]. Because retinal pigment epithelial cells are also damaged, fundus autofluorescence typically shows a hypofluorescent center surrounded by ring-shaped hyperfluorescent lesions [8]. Not only direct sungazing but also other factors, such as environmental conditions including snow, sand, water, and glass, may exacerbate retinal injuries by reflecting light [5,9]. Here, we present a rare case of solar maculopathy associated with reflected sunlight during garden tiling without protective glasses.

## Case presentation

**Figure 1:**
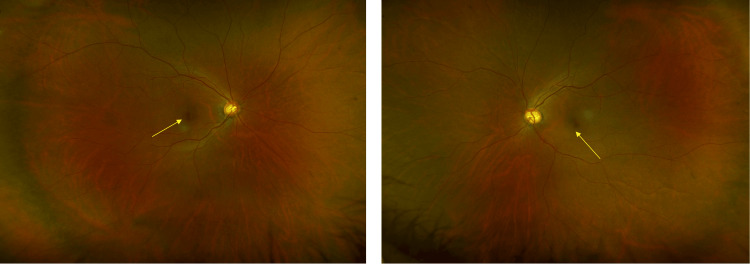
Color fundus photographs at the first visit. Mild color changes were present around the fovea (arrows), and large cupping was present in both eyes. The cup-to-disc ratios were 0.7 OD and 0.8 OS. The cup-to-disc ratio of the left was slightly larger than the right, but the rim color was not pallor in the left.

A 64-year-old woman presented with visual field defects in both eyes during a comprehensive medical checkup and was suspected of macular degeneration at a local eye clinic. She was referred to our hospital three years ago for further examination. She noticed mildly blurred vision and photophobia in both eyes six months earlier. Her best-corrected visual acuities were 1.2 in the right eye (OD) and 1.2 in the left eye (OS). Intraocular pressure was 17 mmHg OD and 17 mmHg OS. Fundus examination revealed mild color changes surrounding the fovea in both eyes and large cupping (Figure 1). The cup-to-disc ratios were 0.7 OD and 0.8 OS. Slit-lamp examination revealed no corneal opacities, no signs of inflammation in the anterior chamber, and mild cataracts in either eye. The vitreous cavities showed neither opacities nor inflammatory signs in both eyes.

**Figure 2:**
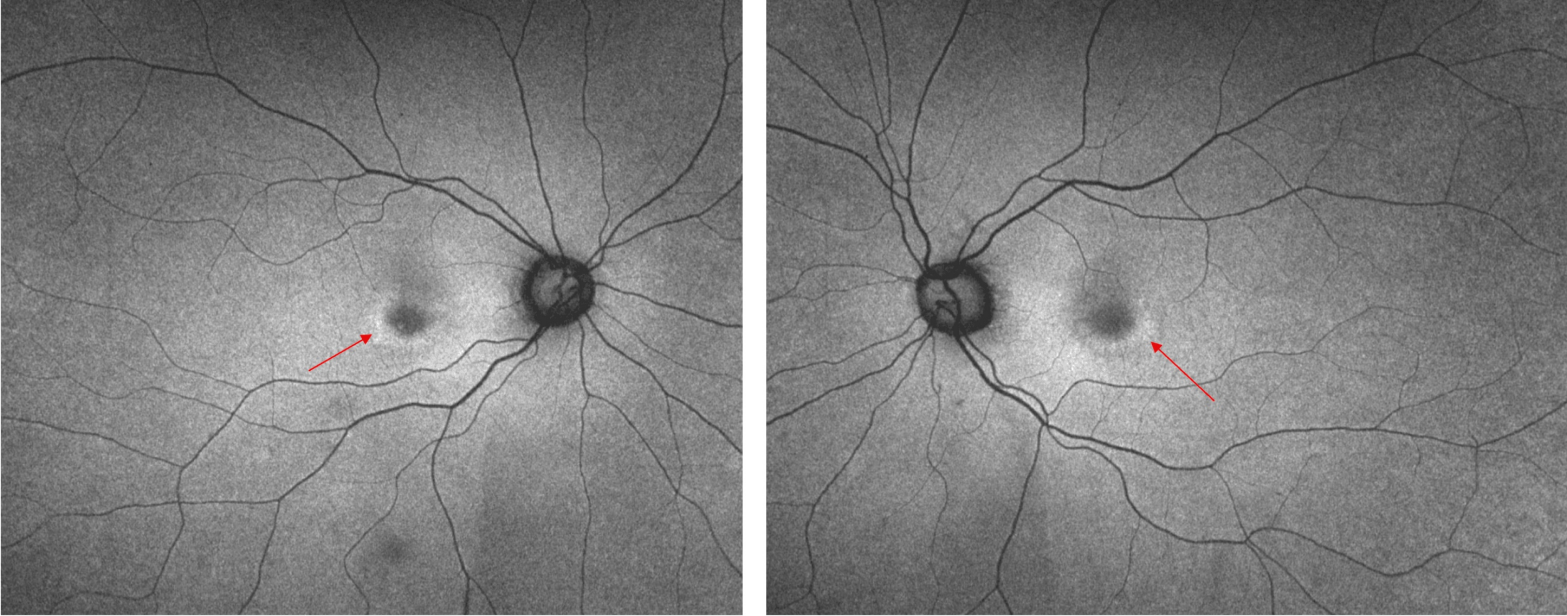
Fundus autofluorescence photographs at the first visit. Ring-shaped hyperfluorescent lesions surrounding the fovea (arrows) were observed.

Fundus autofluorescence revealed ring-shaped hyperfluorescent lesions surrounding the fovea in both eyes (Figure 2). The ring-shaped lesions corresponded to those found in the color fundus photographs.

**Figure 3:**
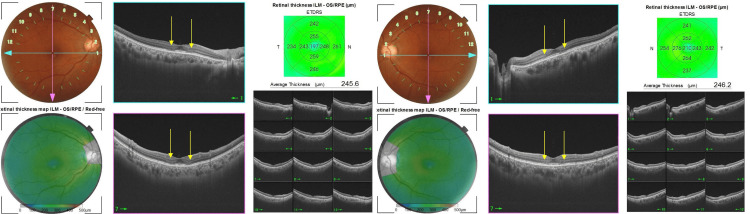
Optical coherence tomography (OCT) findings at the first visit. Disruptions of the ellipsoid zones (EZ) surrounding the fovea were found in both eyes (arrows). Outer retinal thinning was also observed in the parafoveal area (arrows).

OCT findings showed disruption of the ellipsoid zones (EZ) at the parafovea in both eyes (Figure 3). The outer retinal lesions found on OCT were consistent with the ring-shaped lesions found on fundus autofluorescence examination. She had no history of retinal disease, including central serous chorioretinopathy or age-related macular degeneration.

**Figure 4:**
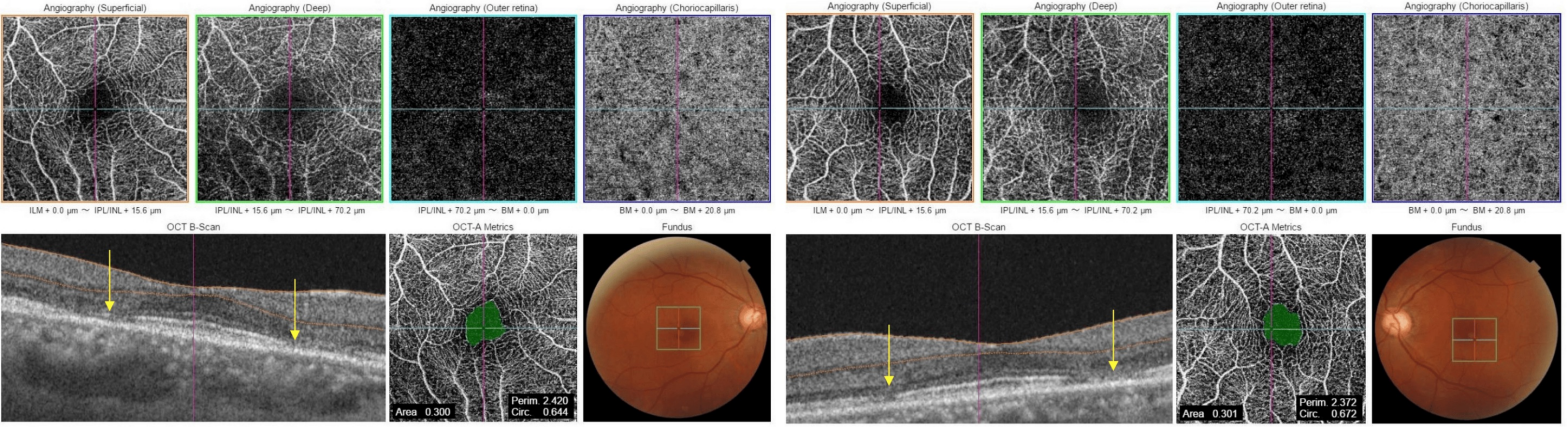
Optical coherence tomography (OCT) angiography examinations at the first visit. No abnormal vessels were found in the macula. The ellipsoid zone (EZ) lines were disrupted, with surrounding areas around the fovea in both eyes (arrows).

**Figure 5:**
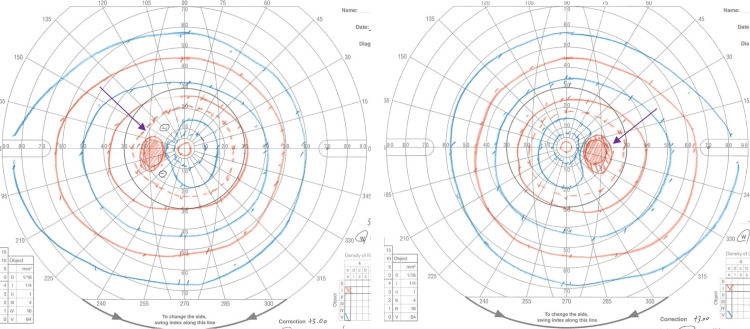
Goldmann perimetry test. Both eyes demonstrated blind spot enlargements without evidence of scotoma (arrows).

**Figure 6:**
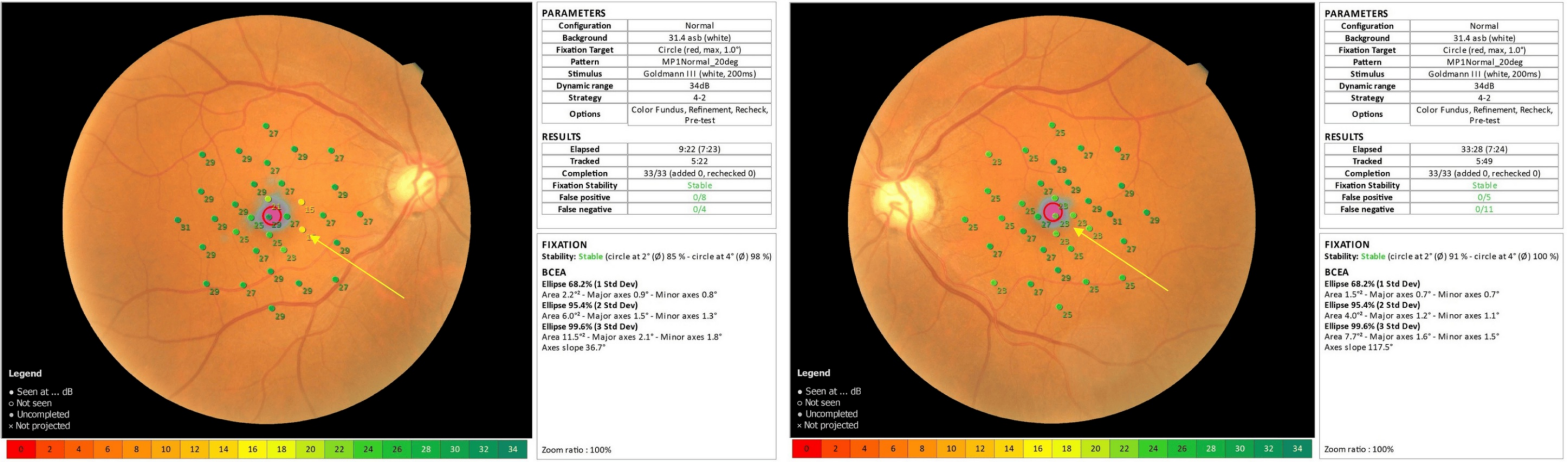
Findings of microperimetry (MP-3) examination. Both eyes have slightly decreased retinal sensitivities surrounding the fovea (arrows).

OCT angiography revealed no abnormalities (Figure 4). The critical fusion frequencies were 41 Hz for the OD and 41 Hz for the OS. The color vision test (panel D15) was normal. Electroretinography (ERG) and multifocal ERG findings were normal. Fluorescein and indocyanine green angiographies revealed no abnormalities. Goldmann perimetry showed blind spot enlargement in both eyes (Figure 5); however, in the microperimetry examination, the retinal sensitivities surrounding the fovea were mildly decreased (Figure 6).

Blood tests showed no remarkable findings, and the serum recoverin antibody test was negative. Serum 5-S-cysteinyldopa levels were within normal limits. Other blood test results are shown in Table 1. No inflammatory reaction was found in the blood test.

**Table 1:** Results of all the laboratory findings. WBC: white blood cells, RBC: red blood cells, Hb: hemoglobin, Ht: hematocrit, Plt: platelets, CRP: C-reactive protein, HbA1c: glycosylated hemoglobin A1c, IgG: immunoglobulin G, IgA: immunoglobulin A, IgM: immunoglobulin M, ACE: angiotensin-converting enzyme, C3: complement component 3, C4: complement component 4, CH50: total hemolytic complement activity (serum complement level), PR3-ANCA: proteinase 3 anti-neutrophil cytoplasmic antibody, MPO-ANCA: myeloperoxidase anti-neutrophil cytoplasmic antibody, β-D-glucan (b-D-glucan): beta-D-glucan, sIL-2R: soluble interleukin-2 receptor, 5-S-CD: 5-S-cysteinyldopa, BUN: blood urea nitrogen, Cre: creatinine, TP: total protein, ALB: albumin, eGFR: estimated glomerular filtration rate, AST: aspartate aminotransferase, ALT: alanine aminotransferase, ALP: alkaline phosphatase, Na: sodium, K: potassium, Cl: chloride, Ca: calcium, P: phosphorus (phosphate), RPR: rapid plasma reagin test, T-SPOT.TB: T-cell spot test for tuberculosis

items	Results	Reference Values	Units
WBC (white blood cells)	5.09	3.30 - 8.60	×10³/mL
RBC (red blood cells)	4.37	3.86 - 4.92	×10⁶/mL
Hb (hemoglobin)	12.4	11.6 - 14.8	g/dL
Ht (hematocrit)	38.7	35.1 - 44.4	%
Plt (platelet)	210	158.0 - 348.0	×10³/mL
CRP (C-reactive protein)	0.09	0.00 - 0.14	mg/dL
HbA1c (glycohemogrobin A1c)	5.8	4.9 - 6.0	%
IgG	872	870 - 1700	mg/dL
IgA	291	110 - 410	mg/dL
IgM	178	35 - 220	mg/dL
ACE (angiotensin-converting enzyme	9.1	8.3 - 21.4	U/L
C3	153	86 - 160	mg/dL
C4	34	17 - 45	mg/dL
serum complement level	37.3	25 - 48	CH50/mL
PR3-ANCA (proteinase 3-anti-neutrophil cytoplasmic antibody)	<0.1	<3.5	U/mL
MPO-ANCA (myeloperoxidase-antineutrophil cytoplasmic antibody)	<1.0	<3.5	U/mL
b-D-glucan	9.6	<20	pg/mL
toxoplasma IgG	<=3	<6	IU/mL
sIL-2R (soluble interleukin-2 receptor)	398	157 - 474	U/mL
5-s-CD (5-s-cysteinyldopa)	2.2	1.5 - 8	nmol/L
BUN (blood urea nitrogen)	14.3	8.0 - 20.0	mg/dL
cre (creatinine)	0.62	0.46 - 0.79	mg/dL
TP (total protein)	7.2	6.6 - 8.1	g/dL
ALB (albumin)	4.2	4.1 - 5.1	g/dL
eGFR (estimated glomerular filtration rate)	74.3		mL/min/1.73m²
AST (asparate aminotransferase)	21	13.0 - 30.0	U/L
ALT (alanine aminotransferase)	16	7.0 - 23.0	U/L
ALP (alkalin phosphatase)	85	38 - 113	U/L
Na	143	138 - 145	mmol/L
K	101	101 - 108	mmol/L
Cl	9.5	8.8 - 10.1	mg/dL
Ca	3.3	2.7 - 4.6	mg/dL
P	108	73 - 109	mg/dL
rapid plasma reagin test	negative		
T-SPOT.TB test	negative		
anti-recoverin antibody	negative		

**Figure 7:**
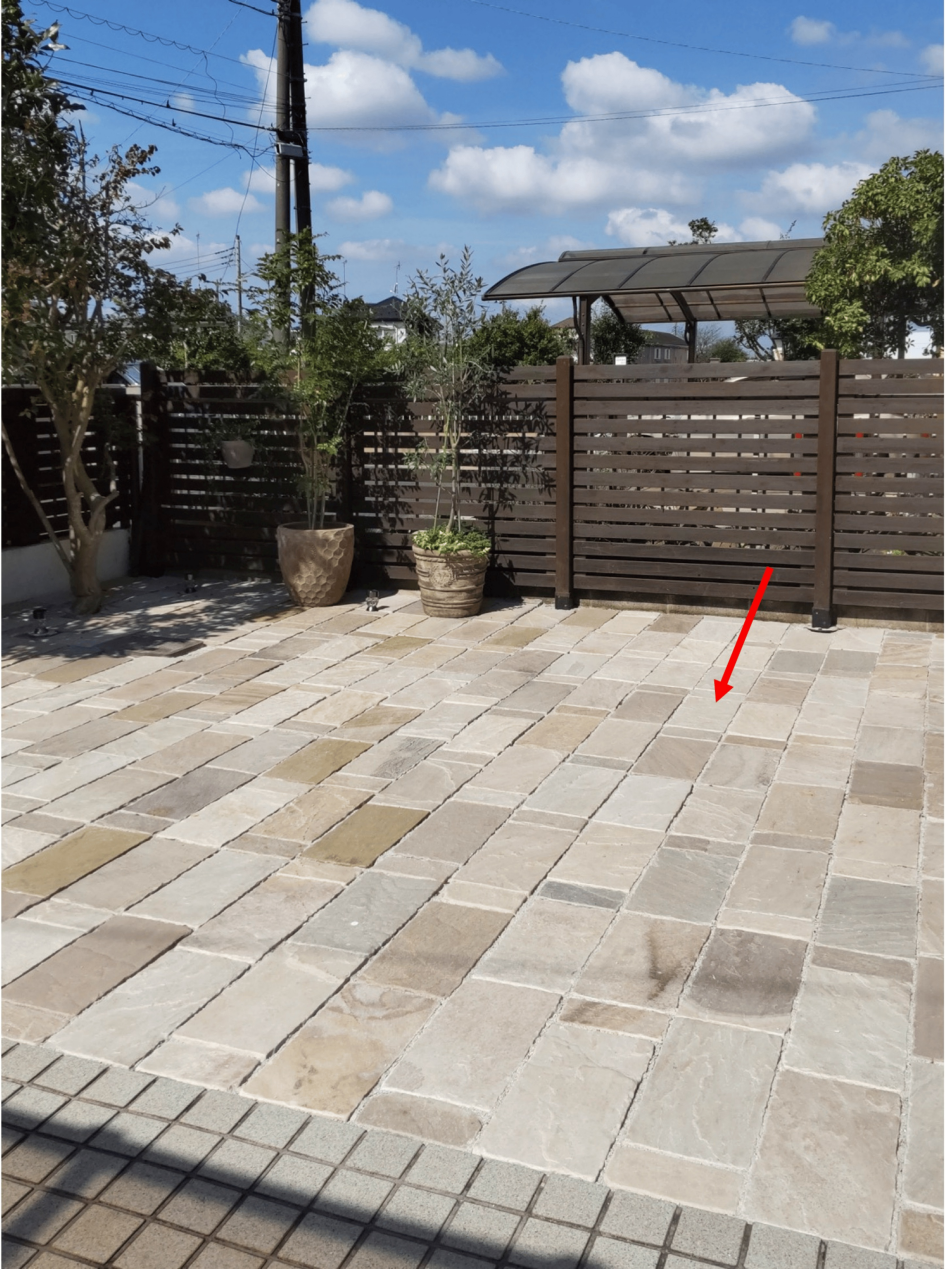
Tiled private garden. The patient tiled her garden alone in August. The average temperature in Japan during that month was approximately 35°C. An arrow indicates the garden tiles.

Screening examinations of the colon, stomach, brain, and breasts revealed no abnormalities. General examination as a whole revealed no signs and symptoms of malignancy in the colon, stomach, brain, and breast. After a detailed medical examination, she reported tilling her garden for over one month during the summer without appropriate sun protection. After tiling, the patient noticed photophobia in both eyes. A photograph of the garden tiles is shown in Figure 7. She wore a large hat and had never seen the sky directly during work. From the picture of the garden, we expected the patient to have been exposed to sunlight reflected from whitish or sand-like tiles for a month during the hot summer in Japan. Based on the one-month history of exposure to reflected sunlight from bright tiles, she was diagnosed with solar maculopathy. No treatment was administered, but we suggested wearing the recommended sunglasses for protection from sunlight. Her symptoms and fundus findings remained unchanged for three years. Informed consent was obtained from the patient for the presentation of pictures of a private garden.

## Discussion

At the initial visit, no history of sunlight exposure was identified; therefore, the final diagnosis of solar maculopathy was established only after several weeks. It had a relatively acute onset, and ERG and multifocal ERG showed no abnormal findings. Therefore, retinitis pigmentosa, cone dystrophy, occult macular dystrophy, and acute zonal occult outer retinopathy were excluded. Furthermore, the patient had no history of using any toxic drugs, including tamoxifen, hydroxychloroquine, chlorpromazine, fingolimod, paclitaxel, digitalis, and icotinib. Thus, drug-induced toxic maculopathy was excluded. In addition, choroidal neovascularization (CNV) was not detected in fluorescein, indocyanine green, and OCT angiographies. Therefore, occult inflammatory conditions induced by CNV were excluded. During the summer, the patient did not go into the pool or the sea. She did not sprinkle water during tiling. So, water reflection did not cause solar maculopathy. She did not sunbathe in the summer, and thus, she had no chance to expose herself directly to sunlight. She has not worked any other job than as a housewife, and thus, she has had no chance to have other occupational exposure, such as welding. Serum anti-recoverin antibody and 5-S-cysteinyldopa tests and cancer screening were negative. Thus, cancer- and melanoma-associated retinopathies were also excluded. Fluorescein and indocyanine green angiographies revealed no abnormal findings; thus, inflammatory diseases such as vasculitis or uveitis were ruled out. Finally, we obtained a relevant history of sunlight exposure prior to symptom onset, allowing us to diagnose solar maculopathy.

In this case, the cup-to-disc ratios were 0.7 OD and 0.8 OS, but the thicknesses of the retinal nerve fiber layer were within the normal ranges: 102 μm OD and 98 μm OS. No glaucomatous changes were observed in the OCT findings. The optic disc anatomy may be related to Mariotte's blind spot enlargements, but is unlikely to be associated with the decrease of retinal sensitivities found in the microperimetry. It is reasonable that the reduction of retinal sensitivities in the microperimetry is due to outer retinal damage involved in solar maculopathy.

OCT and autofluorescence reveal characteristic findings of solar maculopathy, that is, outer retinal changes due to damage of the photoreceptor outer segments and hyper- or hypo-autofluorescence, indicating injury to retinal pigment epithelial cells [8].

In the early phase of phototoxic maculopathy, typical OCT findings include outer retinal hyperreflectivity in the EZ and retinal pigment epithelial cells [7,10,11]. In the chronic phase of severe cases, phototoxic cell death causes outer retinal thinning and EZ disruption [7,12]. Outer retinal holes are a characteristic sign of solar maculopathy and persist in 80% of cases [7,13]. In the present case, the OCT and autofluorescence findings were consistent with those of solar maculopathy. However, these findings are not unique to solar maculopathy; thus, a clear history of sunlight exposure is required for a correct diagnosis.

Several cases of solar maculopathy secondary to reflected sunlight have been reported [5,14]. In the literature, three reported cases of solar maculopathy occurred in environments with surrounding snow or sand. Because snow and sand surfaces have high albedo, which is the ratio of reflected incident light or radiation [9,15,16], these environments, such as snow-covered mountains or sandy beaches, increase the risk of developing solar maculopathy.

Garden tiles in the patient’s private garden appeared to have sand-like surfaces and may have high albedo. One month of reflected sunlight exposure from these tiles during summer, without protective glasses, is thought to have been sufficient to induce outer retinal damage, leading to the development of solar maculopathy. The episode of sunlight exposure was unusual, making the diagnosis challenging; however, ophthalmologists should be aware of the potential of solar maculopathy from reflected sunlight during routine outdoor work.

## Conclusions

This is a rare case of solar maculopathy caused by sunlight reflected from garden tiles. Individuals should wear protective glasses with appropriate filters when working outdoors where reflected sunlight may be intensified.
